# A Fifty Years’ Resume—Observations as to the Kidney of Pregnancy

**Published:** 1917-06

**Authors:** I. L. Van Zandt

**Affiliations:** Fort Worth, Texas


### A FIFTY YEARS' RESUME—OBSERVATIONS AS TO THE KIDNEY OF PREGNANCY.

BY I. L. VAN ZANDT, M. D., FORT WORTH, TEXAS.

The paper begins with a case of edema in a woman eight months pregnant, seen in December, 1866. The edema was so intense that the vulvo-vaginal mucous membrane protruded between the labia majora as two bladders of water, so tense that on the first lancet puncture the water spurted some inches. This patient died a few weeks after confinement, as did most others such at that time.

In 1870 the author discovered that cream of tartar and tincture iron had a distinctly curative effect, relieving some cases when seemingly in desperate conditions.

About 1890 he read a paper on "Edema Gravidarum" before the North Texas Medical Association, calling attention to the fact that these cases were most prevalent in winter and early spring. In 1892, before the same association, he exhibited a chart showing the distribution by months of fifty cases of puerperal convulsions. These also showed a preference for the same seasons, absolutely with regard to multiparae, but among primiparae there were some exceptions. From this he concluded that cold weather was the etiologic factor, and advised that the pregnant woman be warmly clad and avoid being chilled. In primiparae he suggested the pressure of the undistended abdominal wall as a possible additional cause.

About eight years ago he began making indican a basis of treatment; since which time he has had no convulsions and has found albumen in only one pregnant woman in his practice. This was associated with indican and disappeared with its disappearance. He suggests that too much meat and too little vegetables in winter may have caused the winter prevalence, and not cold, *per se*.

He gives the views of Metchnikoff, Caille and Wm. Hanna Thomson as to the deleterious effects of intestinal putrefaction as shown by indicanuria, then concludes as follows:

"My own limited experience suggests this line of thought. Albuminuria results from a traumatism of the kidney, caused by the passage through it of irritating matter. This irritating matter in a large per cent of cases is the resultant of intestinal putrefaction, indicated by indicanuria.

"The vulnerability of kidneys is very variable in degree, some showing albumen with little indican, while others show no albumen with indican intensely marked.

"Hence the conclusion—to prevent kidney troubles of the pregnant woman (and others), keep the intestinal canal clean."

